# Not just minor wild edible forest products: consumption of pteridophytes in sub-Saharan Africa

**DOI:** 10.1186/1746-4269-10-78

**Published:** 2014-12-22

**Authors:** Alfred Maroyi

**Affiliations:** Medicinal Plants and Economic Development (MPED) Research Centre, Botany Department, Faculty of Science and Agriculture, University of Fort Hare, Private Bag X1314, Alice, 5700 South Africa

**Keywords:** Edible pteridophytes, Fern and fern allies, Indigenous knowledge, Sub-Saharan Africa

## Abstract

**Background:**

Gathering of wild edible plant resources by people in sub-Saharan Africa is discussed with reference to pteridophytes, which is an ancient plant group. Pteridophytes are crucial to food diversity and security in sub-Saharan Africa, although they are notably neglected as a result of inadequate research and agricultural development. Current research and agricultural development agenda still appear to focus on the popular and commonly used food crops, vegetables and fruits; ignoring minor and underutilized plant species such as pteridophytes which have shown significant potential as sources of macro and micro nutrients required to improve the diet of children and other vulnerable groups in sub-Saharan Africa. Documentation of edible pteridophytes is needed to reveal the importance of this plant group in the region and the associated indigenous knowledge about them; so that this knowledge can be preserved and utilized species used to combat dietary deficiencies as well as improve food security in the region. The aim of this study is to present an overview of food value of pteridophytes in sub-Saharan Africa using available literature and to highlight their potential in addressing dietary deficiencies in impoverished communities in the region.

**Methods:**

This study is based on review of the literature published in scientific journals, books, reports from national, regional and international organizations, theses, conference papers and other grey materials obtained from libraries and electronic search of Google Scholar, ISI Web of Science and Scopus.

**Results:**

A total of 24 taxa belonging to 14 genera and 11 families are used in sub-Saharan Africa as fodder and human food. *Pteridium aquilinum* (L.) Kuhn is the most common edible pteridophyte in sub-Saharan Africa, used as human food in Angola, Cameroon, DRC, Gabon, Madagascar, Nigeria and South Africa, followed by *Ophioglossum reticulatum* L. (South Africa, Swaziland and Zanzibar), *Ceratopteris thalictroides* (L.) Brongn. (Madagascar and Swaziland), *Diplazium sammatii* (Kuhn) C.Chr. (DRC and Nigeria), *Nephrolepis biserrata* Sw. (DRC and Nigeria) and *Ophioglossum polyphyllum* A. Braun (Namibia and South Africa). The majority of edible pteridophytes are eaten as vegetables or potherbs (66.7%), with some eaten raw or as salad or edible rhizomes (12.5% each). Literature search revealed that some of the documented pteridophytes have high macro and micro nutrient content comparable to recommended FAO/WHO daily nutrient intake from conventional food crops and vegetables.

**Conclusion:**

This study demonstrated the capability of literature research to reveal traditional knowledge on edible pteridophytes in sub-Saharan Africa from dispersed primary ethnobotanical data. Findings from this study suggest that edible pteridophytes could make an important contribution to provision of macro and micro nutrients to the sub-Saharan African population. This study also provided evidence of the importance of pteridophytes as food sources, and can therefore, used to enhance food security in the region by complementing the major food crops, vegetables and fruits.

## Background

Pteridophytes existing today represent ancient plant species which appeared about 300 million years ago in the late Devonian period [1]. Pteridophytes, often referred to as “ferns and fern allies” are vascular plants that produce neither flowers nor seeds, but reproduce and dispersed via spores. The ferns and fern allies do not form a monophyletic group [2] and pteridophyta as a taxonomic group is now regarded as made up of two classes, Lycopodiophyta (fern allies) and Pteridophyta (true ferns) [2, 3]. The Lycopodiophyta is represented by only five relict genera (*Isoetes, Lycopodium, Phylloglossum, Selaginella* and *Stilites*) [3]. The Pteridophyta are much more diverse than the Lycopodiophyta, showing great range of form and cosmopolitan in distribution [3]. Lycopodiophyta and Pteridophyta is a small group of about 12000 species [3, 4], with some species gathered in the wild for food and medicine, and others cultivated as food and ornamental plants. Gathering of wild edible plants persists in many rural communities in sub-Saharan Africa [5–10]. Local people in western Sahel region in sub-Saharan Africa, for example, have been reported to depend on a number of wild edible plants and this dependency increases during drought conditions [11]. Plants collected from the wild are used to supplement the diet of these local communities in sub-Saharan Africa which is based on rainfed cultivation of staple foods such as cassava, maize, millet, sorghum and wheat. The diversity of wild edible plants are known to offer a varied diet to rural communities [10] and wild foods are usually available at times of food shortage [7], and can be of critical importance in livelihood and survival strategies for rural households and communities.

Edible ferns are some of the most common wild food plants collected by people around the world [12–18]. The fern stems, rhizomes, leaves, young fronds and shoots and sometimes the whole plants are used for food [18]. Over time, interest in the food uses of pteridophytes has been sporadic, especially in China [17, 18], Hawaii [13, 16], Japan [19] and Nigeria [15]. Although pteridophytes continue to play an important role in the sub-Saharan Africa, there is a dearth of information on wild edible pteridophytes gathered from the wild in the region. Both surveys and in-depth ethnobotanical studies are needed to adequately record the wealth of cultural and ethnobotanical knowledge on pteridophytes held by different ethnic groups in the region. According to Pieroni [20], evaluation of plant species used in different cultural contexts is necessary in order to infer cultural components related to food acceptance and phytochemical constituents that influence the popularity of edible plants. Plant resources have gained prominence in sub-Saharan Africa as a natural asset through which communities derive food, enabling particularly poor families to achieve self-sufficiency. Documentation of use patterns of pteridophytes across sub-Saharan Africa is of relevance in understanding the importance of this ancient plant group to livelihood strategies of different ethnic groups. This is particularly important for this ancient evolutionary lineage which could potentially become extinct if harvested non-sustainably. The aim of this study is to present an overview of food value of pteridophytes in sub-Saharan Africa using available literature and to highlight their potential in addressing dietary deficiencies in impoverished communities in the region.

## Research methods

Information on wild edible pteridophytes in sub-Saharan Africa was collated. Available references or reports on edible pteridophytes in sub-Saharan Africa were consulted from published scientific journals, books, reports from national, regional and international organizations, theses, conference papers and other grey materials. Literature was searched on international online databases such as ISI Web of Science, Scopus and Google Scholar using specific search terms such as “edible ferns”, “edible fern allies”, “edible pteridophytes”, “wild edible ferns”, “wild edible fern allies” and “wild edible pteridophytes”. References were also identified by searching the library collections of the University of Fort Hare, South Africa. All plant scientific names, plant families and plant authorities were verified using internet sources such as the International Plant Name Index (http://www.ipni.org), the Missouri Botanical Garden’s Tropicos Nomenclatural database (http://www.tropicos.org) and the Royal Botanic Garden and Missouri Botanic Garden plant name database (http://www.theplantlist.org). For each species, data was also collected from literature on countries in which the species are utilized, use(s), edible parts and mode of preparation of the species. Literature search was also done to document the nutritional value of pteridophytes consumed as human food in sub-Saharan Africa.

## Results and discussion

### Pteridophytes diversity

A total of 24 taxa belonging to 14 genera and 11 families are used in sub-Saharan Africa as fodder and human food (Table 1). Leaves of *Dryopteris wallichiana* (Spreng) Hyl., *Nephrolepis biserrata* (Sw.) Schott and *Ophioglossum grande* L. are used as fodder for goats, sheep and other small ruminants in Nigeria [15, 21], while the rest of the species are used as human food. All reported species are indigenous to the region, with *Pteridium aquilinum* (L.) Kuhn as the most widespread fern, occurring mainly as a cosmopolitan weed with an almost worldwide distribution apart from mountainous, desert and arctic areas [22]. Plant families with the highest number of food plants are Ophioglossaceae, represented by seven species, followed by Athyraceae represented by three species; and Davalliaceae, Dennsteadtiaceae, Marsileaceae, Pteridaceae and Thelypteridaceae represented by two species each. Athyraceae, Dennsteadtiaceae, Ophioglossaceae, Pteridaceae and Thelypteridaceae are among the most species-rich pteridophyte families, represented by at least 80 species [2, 23]. The rest of the families are represented by one species each, as shown in Table 1. The genera with highest number of species are *Ophioglossum* represented by six species, followed by *Diplazium* represented by three species; and *Ceratopteris*, *Marsilea* and *Nephrolepsis* represented by two species each. *Ceratopteris*, *Diplazium*, *Marsilea, Nephrolepsis* and *Ophioglossum* have the highest diversity of species probably because these are large genera worldwide, characterized by at least five species each [23].

**Table 1:** **List of pteridophytes used as fodder and human food in sub-Saharan Africa**

Species, family name	Country, vernacular name	Main uses and references
*Blotiella glabra* (Bory) R.M.Tryon; Dennsteadtiaceae	DRC: Asaha, oheyi yasi, oheyi	Young leaves eaten raw or cooked as leafy vegetable [24]
*Botrychium lanuginosum* Wall. ex Hook et Grev.; Ophioglossaceae	Nigeria: Grape fern, iya, oziza ato	Young leaves eaten as green vegetable [15]
Ceratopteris cornuta (P. Beauv.) Lepr.; Pteridaceae	Liberia: Water lettuce	Cultivated and eaten as a leafy vegetable [14, 25]
*Ceratopteris thalictroides* (L.) Brongn.; Pteridaceae	Madagascar	Leaves eaten as salad or cooked as vegetable [26]
	Swaziland	Leaves eaten as leafy vegetable [27]
*Christella dentata* (Forssk.) Brownsey & Jermy; Thelypteridaceae	DRC: Anole	Young leaves cooked as leafy vegetable [24]
*Cyclosorus gongylodes* (Schkuhr) Link; Thelypteridaceae	Gambia	Leaves eaten as leafy vegetable [28]
*Cyathea manniana* Hook.; Cyatheaceae	DRC: Oyaele	Young leaves cooked as leafy vegetable [24]
*Diplazium esculentum* (Retz.) Sw.; Athyraceae	Nigeria: Akwukwo nni, vegetable fern	Young leaves eaten as vegetable with yam (*Dioscorea* spp.) [15]
*Diplazium proliferum* (Lam.) Thouars; Athyriaceae	Madagascar	Young still enrolled fronds (croziers, fiddleheads) are eaten as a cooked vegetable [29]
*Diplazium sammatii* (Kuhn) C.Chr.; Athyraceae	DRC: Andole, aneke	Young leaves are cooked as leafy vegetable [24]
	Nigeria: Nyama idim	Young still enrolled fronds (croziers, fiddleheads) are eaten as a cooked vegetable [30]
*Dryopteris wallichiana* (Spreng) Hyl.; Dryopteridaceae	Nigeria: Mountain wood fern, mbabe, ire, aja nmuo	Leaves are used as fodder for goats and sheep [15]
*Lomariopsis* sp.; Lomariopsidaceae	DRC: Asaha	Young leaves cooked as condiment [24]
*Marsilea minuta* L.; Marsileaceae	Gambia	Tender leaves eaten as a potherb [31]
*Marsilea minuta* L.; Marsileaceae	Senegal	Tender leaves eaten as a potherb [31]
*Nephrolepis biserrata* (Sw.) Schott; Davalliaceae	DRC: Asaha, likekele	Young leaves are cooked as condiment or leafy vegetable [24]
	Nigeria	Leaves used as fodder for goats and other ruminants [21]
*Nephrolepis cordifolia* (L.) Presl.; Davalliaceae	Nigeria: Erect swordfern, nma ozo	Rhizomes are cleaned and boiled with salt and water and eaten as food [15]
*Ophioglossum grande* L.; Ophioglossaceae	Nigeria: Ribbon fern, achu, tsage	Young fronds used as fodder for goats [15]
*Ophioglossum lusoafricanum* Prantl; Ophioglossaceae	Swaziland: Adder's tongue, sankunshane, sankuntjane, shucelane	Edible leaves [27]
*Ophioglossum ovatum* Bory; Ophioglossaceae	Madagascar, Antandroy tribe	Leaves eaten as vegetable [32]
*Ophioglossum polyphyllum* A. Braun; Ophioglossaceae	Namibia	Used as famine food when few other plants are available but the species is not popular or well known [33]
	South Africa: isiNkuntshane, isiNdletshane	Leaves eaten as leafy vegetable [6, 34]
*Ophioglossum reticulatum* L.; Ophioglossaceae	South Africa: Adder-tongue fern	Leaves eaten as vegetable [35]
	Swaziland: Adder's tongue	Leaves eaten as leafy vegetable [27]
	Zanzibar, Tanzania	Leaves eaten as salad or cooked as vegetable [36]
*Ophioglossum vulgatum* L.; Ophioglossaceae	Nigeria	Leaves eaten as leafy vegetable [15]
*Pteridium aquilinum* (L.) Kuhn; Dennsteadtiaceae	Angola	Several tribes eat the leaves [32]
	Cameroon	Consumed on a regular basis together with *Vernonia amygdalina* Delile and *Triumfetta rhomboidea* Jacq. [32]
	DRC: lilele, isili	Immature fronds cooked as condiment or vegetable [24, 37]
	Gabon	Young still enrolled fronds (croziers, fiddleheads) are eaten [32]
	Madagascar	Rhizome is eaten [32]
	Nigeria: Eastern bracken, turkey foot fern, ogoni, ukwunnume	Decoction of rhizome drunk as herbal health tea, young fronds eaten as vegetables [15]. Young still enrolled fronds (croziers, fiddleheads) are eaten as vegetable by the tribal people [38]
	South Africa: Adelaarsvaring, brackern fern, eagle fern, umbewe, umhlashoshana	South Africa: Young fronds and rhizomes are cooked and eaten by the Zulu and Tswana [39]
*Stenochlaena tenuifolia* (Desv.) T.Moore; Blechnaceae	Madagascar	The young fronds (croziers, fiddleheads) are eaten as vegetable [40]

**Figure 1:**
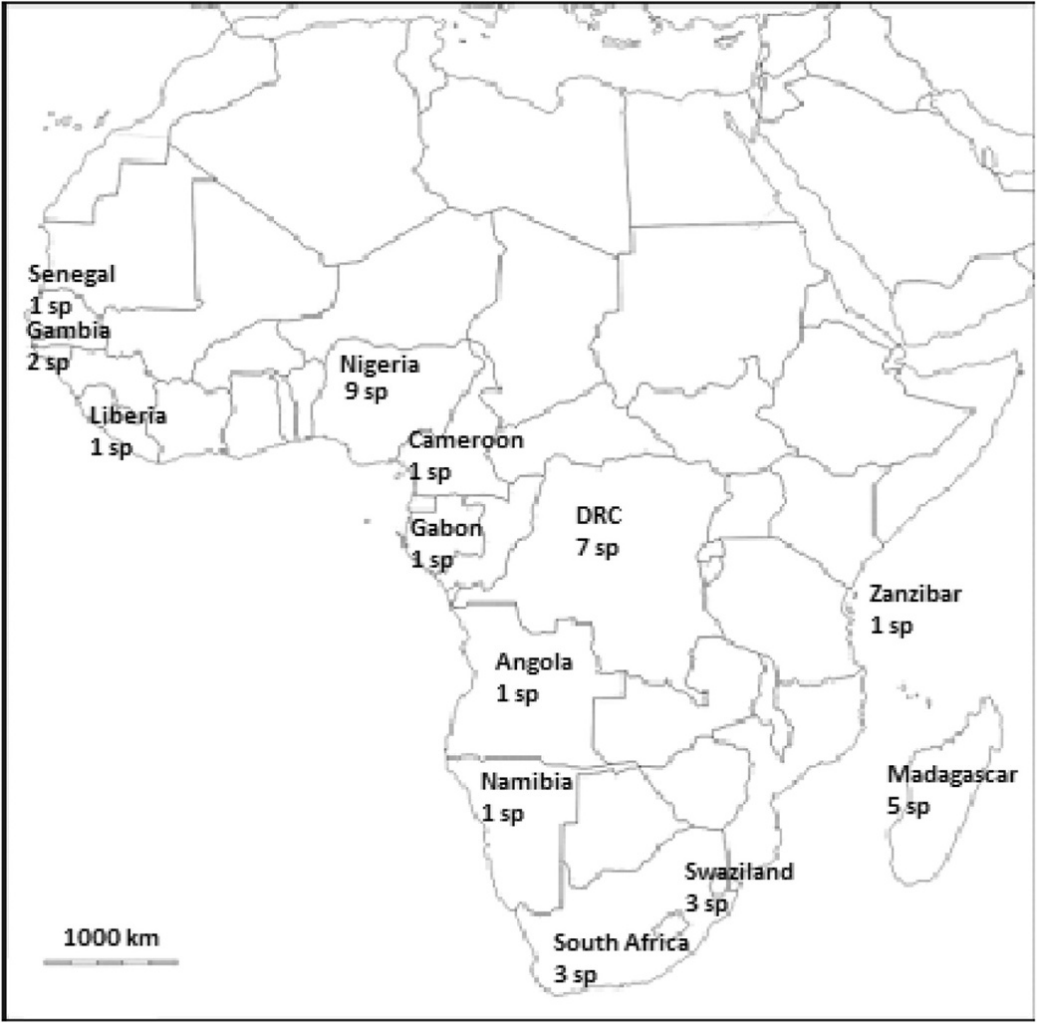
**Pteridophyte species used as fodder and human food in sub-Saharan Africa.**

Edible pteridophytes have been recorded from east, central, south and west Africa (Figure 1). Most of the ethnobotanical data on edible pteridophytes have been reported in DRC and Nigeria (Table 1). In Nigeria, seven species: *Botrychium lanuginosum* Wall. ex Hook et Grev, *Diplazium esculentum* (Retz.) Sw., *Diplazium sammatii* (Kuhn) C.Chr., *Nephrolepis cordifolia* (L.) Presl., *Ophioglossum vulgatum* L. and *Pteridium aquilinum* (L.) Kuhn are utilized as human food [15, 30, 38]. Seven edible taxa have been reported in DRC, which include *Blotiella glabra* (Bory) R.M.Tryon, *Christella dentata* (Forssk.) Brownsey & Jermy, *Cyathea manniana* Hook., *Diplazium sammatii*, *Lomariopsis* sp., *Nephrolepis biserrata* (Sw.) Schott and *Pteridium aquilinum* [24, 37]; and five species among them, *Ceratopteris thalictroides* (L.) Brongn., *Diplazium proliferum* (Lam.) Thouars, *Ophioglossum ovatum* Bory, *Pteridium aquilinum* and *Stenochlaena tenuifolia* (Desv.) T. Moore have been reported in Madagascar [26, 29, 32, 40]. Three edible species have been reported in South Africa, which include *Ophioglossum polyphyllum* A. Braun [6, 34], *Ophioglossum reticulatum* L. [35] and *Pteridium aquilinum* [39]. Three edible species have been reported in Swaziland, which include *Ceratopteris thalictroides*, *Ophioglossum lusoafricanum* Prantl and *Ophioglossum reticulatum* [27]. *Cyclosorus gongylodes* (Schkuhr) Link and *Marsilea minuta* L., are the only two edible species reported in Gambia [28, 31], *Pteridium aquilinum* reported in Angola, Cameroon and Gabon [32], *Ceratopteris cornuta* (P. Beauv.) Lepr. reported in Liberia [14, 25], *Ophioglossum polyphyllum* A. Braun reported in Namibia [33], *Marsilea minuta* L. reported in Senegal [31] and *Ophioglossum reticulatum* reported in Zanzibar [36].

Pteridophytes occur throughout sub-Saharan Africa, particularly in rainforests and afromontane forests of east, central, south and west Africa, making the plant group effective and reliable source of human food. However, due to lack of direct evidence for pteridophyte use from other countries, particularly in north and central Africa, the importance of pteridophytes can only be inferred from ethnobotanical studies done in Angola, Cameroon, DRC, Gabon, Gambia, Liberia, Madagascar, Namibia, Nigeria, Senegal, South Africa, Swaziland and Zanzibar (Figure 1). Although findings from these countries about edible pteridophytes are diverse in terms of utilized species, the findings may not apply directly to all countries in the sub-Saharan African region. It is clear that more detailed national level studies should be undertaken, particularly in north and central Africa where documentation on pteridophyte use is scanty or missing.

### Pteridophytes collection and parts consumed

**Figure 2:**
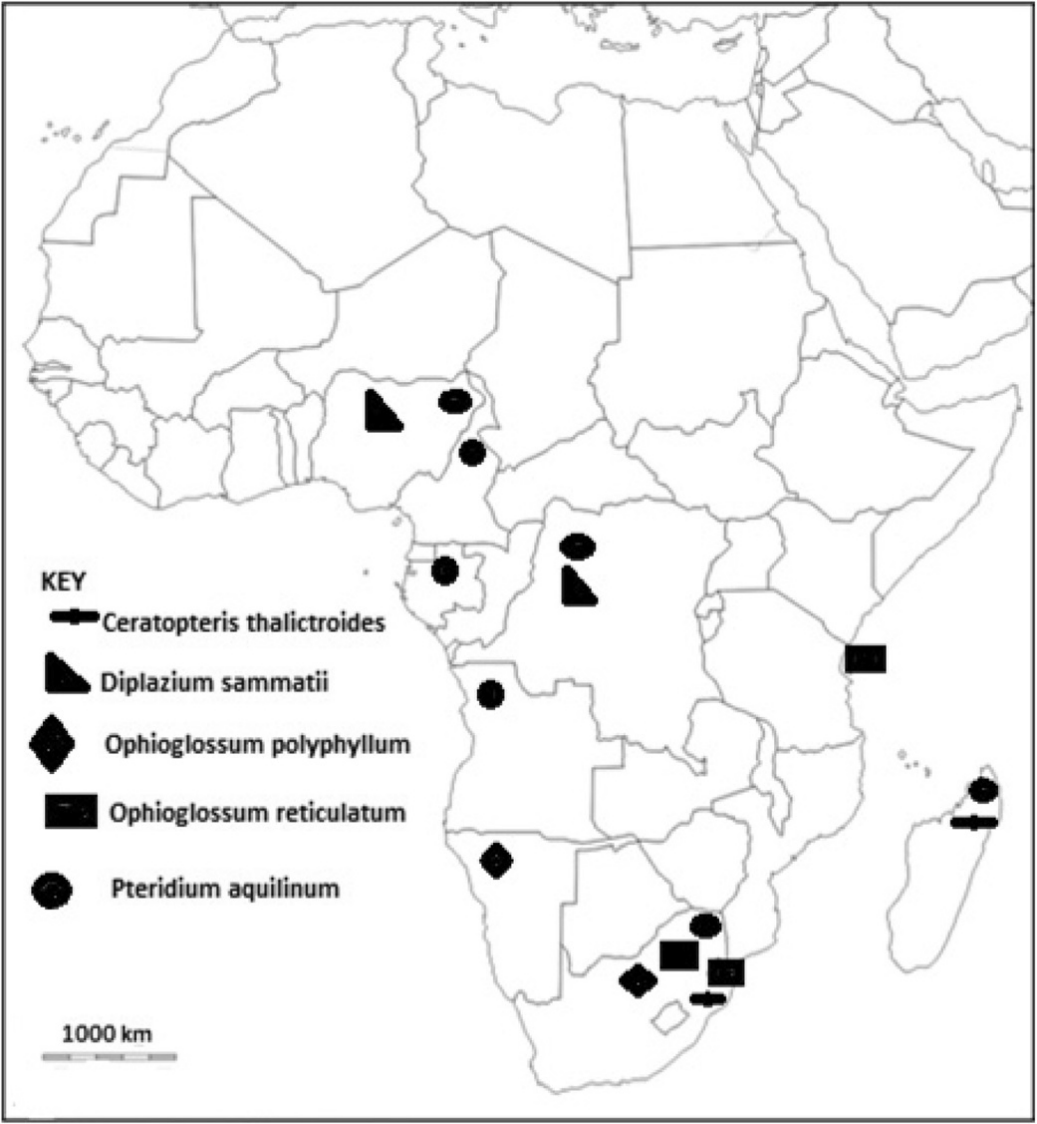
**Distribution of five common edible pteridophytes in sub-Saharan Africa.**

*Pteridium aquilinum* is the most widely used pteridophyte as a source of human food (Figure 2). It is used as food in Angola, Cameroon, DRC, Gabon, Madagascar, Nigeria and South Africa. *Ophioglossum reticulatum* is utilised as human food in eastern and southern Africa, that is, in South Africa, Swaziland and Zanzibar (Figure 2). *Ceratopteris thalictroides* is used as human food in Madagascar and Swaziland, *Diplazium sammatii* is utilized as human food in DRC and Nigeria; and *Ophioglossum polyphyllum* is used as human food in Namibia and South Africa (both countries in southern Africa) (Figure 2). Despite its worldwide distribution, the range of basic applications of *Pteridium aquilinum* are similar across its range of distribution, and it is mainly used as a food source [22]. In Japan and Korea, young shoots of *Pteridium aquilinum* are an important dietary element [22, 41]. The shoots are soaked for a day in water and ashes, then steamed or boiled and eaten as a vegetable or soup [41]. Research by Liu et al. [18] revealed that the leaves and rhizomes of *Pteridium aquilinum* are consumed by billions of people in the world. The same authors argued that *Pteridium aquilinum* is part of the diet of many poor people in south Pacific islands to northern and temperate prairies because of its wide distribution and easy accessibility. *Ophioglossum* are widely eaten in Asia, with *Ophioglossum reticulatum* used as a vegetable in India and Indonesia [17], while *Ophioglossum polyphyllum* is consumed as a vegetable in south central Tibet [17]. Research by the same authors, revealed that *Ophioglossum polyphyllum* is eaten in summer as a vegetable but also dried and stored for later consumption in winter. *Ceratopteris thalictroides* is commonly eaten throughout south east Asia, for example, in Malaysia and Japan where it is an established luxury vegetable [26]. According to Bassey et al. [30], various parts of *Diplazium sammatii* are consumed by various people throughout the world. The same authors argued that *Diplazium sammatii* is not an emergency or primitive food type. This assertion by Bassey et al. [30] correlates strongly with observations made by Pieroni [41] that eating food from the wild is not simply an essential response in times of famine or food shortages or an easy way to obtain primary nutrients, but more often a complex evolutionary process involving different aspects of the relationship between humans and their natural environment.

**Figure 3:**
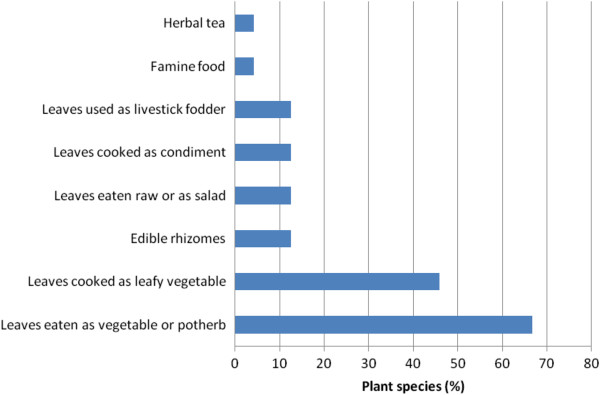
**Characteristics of plant parts used.** Most species are reported in more than one use category.

Young leaves or enrolled fronds, often referred to as crosiers or fiddleheads are the primary pteridophyte food sources in sub-Saharan Africa (70.8%*), followed by leaves (50%*) and rhizomes (16.7%*) (Table 1). (*Some plant parts are reported in more than one plant part category). The majority of pteridophytes are eaten as vegetables or potherbs (66.7%), followed by leaves cooked as leafy vegetables (45.8%), leaves cooked as condiment, leaves eaten raw or as salad, edible rhizomes and leaves fed to livestock (12.5% each), herbal tea and famine food (4.2% each) (Figure 3). When a rhizome is eaten, leaf bases are removed, cleaned and peeled. Pteridophytes consumed as leafy vegetables or potherbs or salads, are usually collected at vegetative stage, when leaves are young and tender. These findings correlates with observations made by Lognay et al. [17] that bundles of freshly uncurled fronds are a common site in the rural markets of the Philippines, Indonesia, Malaysia and New Guinea.

### Suitability of pteridophytes as food sources

Some pteridophytes used as food sources in sub-Saharan Africa, for example, *Diplazium esculentum* [42], *Diplazium sammatii* [30], *Nephrolepis biserrata* [43, 44], *Nephrolepis cordifolia* [21] and *Ophioglossum polyphyllum* [17], have high nutritional value in comparison to the FAO/WHO nutrient standards [45] (see Table 2). These five edible pteridophytes are important sources of macro and micro nutrients which are important for the maintenance of good health and prevention of diseases. The calorific value of 408.5 kcal kg^-1^ for *Diplazium sammatii* and 3413 kcal kg^-1^ for *Diplazium esculentum* (Table 2), compare favourably to 210–1340 kcal kg^-1^ reported for common vegetables (broad beans, cabbage, cauliflower, lettuce, spinach) and common fruits (apple, lichi, mango, pawpaw) reported by Seal [42]. The carbohydrate and protein content of the edible pteridophytes shown in Table 2 is also higher than the carbohydrate and protein content of common vegetables and fruits obtained by Seal [42]. All the five edible pteridophytes have high levels of minerals in comparison to mineral content of common food crops, vegetables and fruits available in the published literature. For example, copper and zinc content of the edible pteridophytes shown in Table 2 is higher than the copper and zinc content of common vegetables and fruits obtained by Seal [42]. Therefore, pteridophytes are an important source of macro and micro nutrients and may be consumed by local communities to enhance nutrient intake or diversify the diet of local communities in sub-Saharan Africa.

**Table 2:** **Nutritional value of edible pteridophytes in sub-Saharan Africa**

Species	Nutritional value													Reference(s)
	kcal kg ^-1^	g kg ^-1^				mg kg ^-1^								
	Energy	Protein	Fat	Fibre	Carbohydrates	Ca	P	K	Na	Mn	Cu	Zn	Fe	
*Diplazium esculentum*	3413	143.8	1.3	38.8	83.9	8730	-	43730	1180	51	26	167	257	[42]
*Diplazium sammatii*	408.5	10.3	11.8	0.4	65.5	1900	7.0	1600	520	-	3.0	4.1	5.5	[30]
*Nephrolepis biserrata*	-	24.5	1.7	14.2	45.9	223.2	-	500	250	100.8	158.8	-	1182	[43, 44]
*Nephrolepis cordifolia*	-	10.3	-	1.6	21.5	26.9	-	141.2	45.5	0.7	7.2	1.1	7.5	[21]
*Ophioglossum polyphyllum*	-	24.5	-	-	-	0.55	4.9	4.18	0.46	27	17	29	888	[17]

Pteridophytes can also play an important role to food security in sub-Saharan Africa, can be utilized during drought periods when few crops are available and their year round availability increases their value as an important food plant group. The data documented in this study can be used in determining which pteridophytes can be preferentially utilized to benefit the overall nutrition of communities who inhabit sub-Saharan Africa. There is uniformity in the use of edible pteridophytes in sub-Saharan Africa, regardless of cultural differences in aspects such as language, history and social organization in general (Table 1, Figure 1). Based on the current baseline study, there is need to do further studies in other countries in the African continent aimed at documenting pteridophytes diversity used as food which at the present appear to be more or less homogenous. The observed similarities in pteridophytes use by different ethnic and cultural groups provide an insight into the possibility that regional groups may have been more closely linked in the past. It is probable that pteridophytes were widely consumed by the pastoralists and hunter-gatherers, historically referred to as the bushmen in sub-Saharan Africa. The oral transmission of traditional knowledge on the uses of pteridophytes by indigenous people has largely been eroded over the centuries due to land fragmentation, migration and improved agricultural activities in favour of improved food crops, vegetables and fruits. Detailed studies across national boundaries are also important in order to find out the possible reasons for similarities in pteridophyte uses. Traditional and cultural knowledge about pteridophytes use should vary, as this indigenous knowledge is not formalized. Detailed comparison of pteridophyte use by different ethnic groups will provide us with a clue to understanding pteridophytes use history, cultural diversity and variations of cultural practices of these ethnic groups.

## Conclusion

This study examined edible pteridophytes, some of them widely distributed in sub-Saharan Africa but whose economic value have not been fully explored. Local people in sub-Saharan Africa use different pteridophytes species as food. Interest in expanding the use of minor and underutilized plant resources such as pteridophytes has been sporadic, especially in rural development initiatives. The uses of pteridophytes outlined in this study including the nutritional information of some of the edible pteridophytes underscore the untapped potential of pteridophytes in meeting basic human needs. An effort should be made to initiate and harness the synergetic relationships that exist between pteridophytes uses and indigenous knowledge of people in sub-Saharan Africa, passed on from generation to generation. This review also demonstrated the capability of literature research to reveal indigenous knowledge by researching and compiling information from the growing body of dispersed primary ethnobotanical data. The core challenge that confronts sub-Saharan Africa is the promotion of edible pteridophytes as an alternative means of addressing and combating dietary deficiencies, thereby improving food security in the region.
